# Sacbrood Virus: A Growing Threat to Honeybees and Wild Pollinators

**DOI:** 10.3390/v14091871

**Published:** 2022-08-25

**Authors:** Ruike Wei, Lianfei Cao, Ye Feng, Yanping Chen, Gongwen Chen, Huoqing Zheng

**Affiliations:** 1 College of Animal Sciences, Zhejiang University, Hangzhou 310058, China; 2 Institute of Animal Husbandry and Veterinary Science, Zhejiang Academy of Agricultural Sciences, Hangzhou 310021, China; 3 Institute of Translational Medicine, Zhejiang University School of Medicine, Hangzhou 310058, China; 4 USDA-ARS Bee Research Laboratory, Beltsville, MD 20705, USA

**Keywords:** sacbrood virus, etiology, epidemiology, transmission, pathogenesis, diagnostics, prevention

## Abstract

Sacbrood virus (SBV) is one of the many viruses that infect both the Western honeybee (*Apis mellifera*) and the Eastern honeybee (*Apis cerana*). Recently, the interspecies transmission of SBV has been discovered, especially among wild pollinators. This newly discovered evolutionary occurrence regarding SBV indicates a much wider host range than previously believed, causing further concern about the future sustainability of agriculture and the resilience of ecosystems. Over the past few decades, vast numbers of studies have been undertaken concerning SBV infection in honeybees, and remarkable progress has been made in our understanding of the epidemiology, pathogenesis, transmission, and manifestations of SBV infection in honeybees and other pollinators. Meanwhile, some methods, including Chinese medicine, have been established to control and prevent sacbrood disease in *A. cerana* in Asian countries. In this review, we summarize the existing knowledge of SBV and address the gaps in the knowledge within the existing literature in the hope of providing future directions for the research and development of management strategies for controlling the spread of this deadly disease.

Honeybees are an essential part of agricultural food production and ecological diversity as they provide critical pollination services for a broad range of the world’s food crops and flowering plants [1]. Nevertheless, over the past decades, elevated honeybee colony losses have been reported in many parts of the world, primarily in the United States and Europe [2,3]. Among the factors that negatively impact bee health, viruses pose one of the major threats to honeybees’ well-being and have caused serious concerns among researchers and beekeepers.

Sacbrood virus (SBV) was the first virus that was identified as having infected honeybees [4], and it continues to be one of the most common viruses found in honeybees worldwide [5,6,7]. Although SBV infection does not usually result in colony losses to the Western honeybee (*Apis mellifera*) [8,9], it is the single greatest threat facing the Eastern honeybee (*Apis cerana*). The catastrophic outbreak of sacbrood disease killed 95–100% of *A. cerana* colonies in different regions of Asian countries [10], such as China, India, Vietnam, Thailand, and South Korea [11,12,13,14,15,16]. As a result, SBV has been extensively studied in these nations. In this review, we summarize the progress of the research relating to SBV to provide a comprehensive foundation in terms of what is currently known about SBV and then identify knowledge gaps that require further research.

## 1. Epidemiology

### 1.1. Symptoms

**Figure 1:**
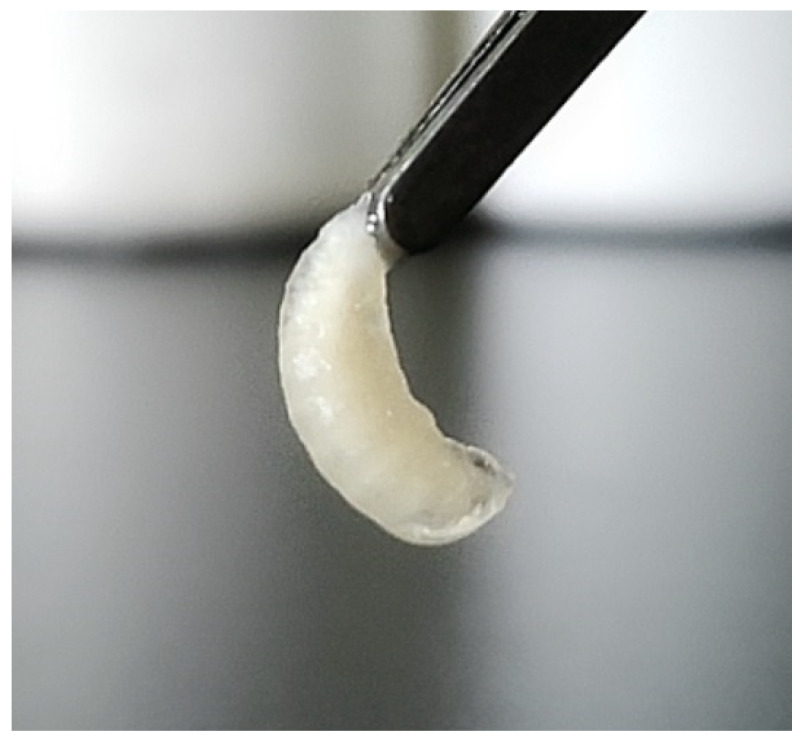
Sacbrood disease of an *Apis cerana* larva (photograph by Xiaoqing Li).

SBV can infect both the brood and adult stages of honeybees’ life cycles, yet larvae about two days old are the most sensitive to the threat it poses [17]. As the disease progresses, SBV particles replicate on a massive scale in larvae and accumulate in the ecdysial fluid between their body and their pouch-like skin, forming a characteristic fluid-filled sac (Figure 1)—hence the name [18]. The larvae cannot shed their skin because of the old leathered cuticle and fail to pupate, meaning there are significantly high mortality levels after the brood cell is capped [19,20]. From illness to death, the color of the body’s surface gradually changes from white to light yellow and then to tan. Dead larvae eventually become dark, brittle, gondola-shaped scales that can be easily removed from the brood cell [21,22,23].

Consequently, dead larvae discovered outside the hives are seen as a symptom of the disease as the ones that died inside the comb are dragged out by the adults. Inside the hive, pointy-headed diseased larvae found on the brood frame stay stretched on their backs, with their heads pointing toward the top of the cell. The diseased and dead larvae appear to take the shape of a small sac or bag when picked up with tweezers [20]. The capping of the diseased larvae is generally caved in and perforated, and they have a few partially or totally uncapped brood cells strewn throughout [20]. The typical characteristics of SBV in *A. mellifera* and *A. cerana* are similar, but the main difference is that the extensive brood removal of *A. mellifera* is not apparent [24], which may be due to their different hygienic behaviors [25].

SBV also causes infection in adult bees but does not cause apparent disease symptoms [26]. SBV-infected adults showed a preference for nectar rather than pollen, resulting in nutritional deficiencies in the honeybee colony and reduced life spans of the honeybees [27,28].

### 1.2. Transmission Routes

The transmission of SBV in honeybee colonies can occur through both pathways, vertical and horizontal transmissions [22,29,30]. SBV infections were identified in the reproductive tissues of the queen and drones, and the virus could be vertically transmitted from mother queens to their offspring, following infection of the queen ovary and eggs [31]. Venereal transmission of SBV was also identified in honeybees, where the virus was transmitted to queens via drone semen during mating or artificial insemination [31,32].

SBV particles left on pollen, honey, and dead larvae have been demonstrated to be infectious for up to four weeks [33,34]. Consequently, the spread of SBV can occur through horizontal transmission by a direct route, such as a food-borne infection. For example, the infection cycle can start with an adult bee collecting virus-contaminated pollen and delivering it to the colony [35]. Subsequently, nurse bees may be infected by exchanging food with contagious adult bees or feeding on virus-contaminated pollen. Moreover, virus particles collect in infected nurse bees’ hypopharyngeal glands. They can disseminate the virus throughout the colony by feeding larvae with glandular secretions and sharing food with other adult bees, especially the queen, who can lay infected eggs. Other healthy nurse bees can also become infected while cleaning the hive and removing the larvae killed by SBV [22]. Contagious foraging bees spread virus particles to plants in surrounding habitats by transmitting the particles from their glandular secretions to pollen burdens as they collect the material. Indeed, spontaneous swarming of bees and the transfer of the honeycomb to different sites by beekeepers might also spread the virus from one hive to new locations and hosts.

Moreover, the ectoparasitic mite *Varroa destructor* plays a critical role in SBV transmission [36]. Many studies have found a significantly higher prevalence of SBV in colonies infested with *V. destructor* mites than those that were free of mite infestation [37,38,39,40]. However, there has been no evidence of SBV replication in *V. destructor*, suggesting that the mites might merely act as mechanical carriers of the virus [38,41].

### 1.3. Prevalence

**Figure 2:**
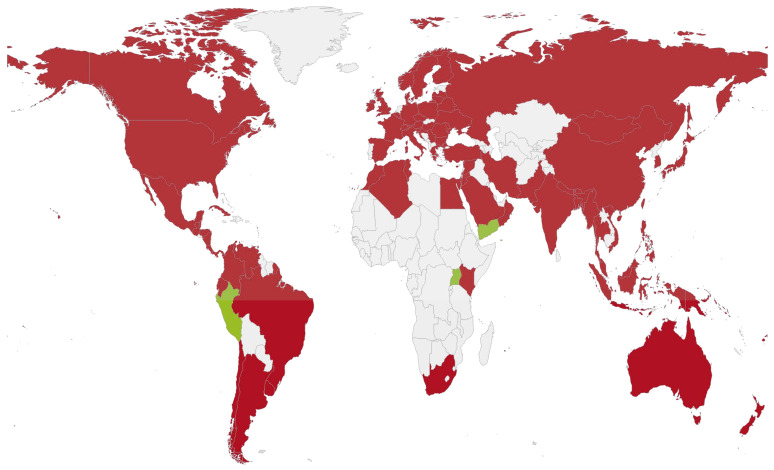
Worldwide distribution of sacbrood virus (SBV). The red color indicates the presence of SBV in the respective regions. The green color indicates regions where previous studies have not reported SBV infection. The gray color indicates that data are not available in these regions.

The worldwide distribution and prevalence of SBV infection are shown in Figure 2 and Table S1. The infection rate of SBV in honeybees has been discovered to be substantially seasonal, with peaks in the spring in both *A. mellifera* and *A. cerana* [42,43,44,45,46]. This can be explained in relation to the higher number of susceptible broods that are reared during the spring, when rich sources of pollen and nectar are available [22]. In addition, the frequently fluctuating temperatures during this season are another factor that contribute to the elevated prevalence and incidence of SBV [47]. When comparisons were made between the honeybee species, the infection rates and loads of SBV were higher in *A. cerana* colonies than in *A. mellifera* colonies [45], confirming that SBV poses a more considerable danger to the former than to the latter.

## 2. Etiology

### 2.1. Genome 

SBV belongs to the genus *Iflavirus* in the family *Iflaviridae* under the order *Picornavirales* and is a small non-enveloped virus with a monopartite and monocistronic positive-stranded RNA genome [48,49].

The genomic RNA of SBV is 8600–8900 nucleotides in length, including an open reading frame that encodes a putative polyprotein of about 2800 amino acids [50]. The genomic structure and protein domain arrangement share common characteristics of the *Iflaviridae* family: the structural proteins are located at the N-terminal portion of the polyprotein in the order of VP2, VP4, VP3, and VP1, and the non-structural proteins are identified as helicase, protease, and RNA-dependent RNA polymerase (RdRp) that are located in the C-terminal portion of the polyprotein [51,52]. However, there are no resolved characteristics on the electron density map of the SBV virion that may be interpreted as VP4 residues [53].

### 2.2. Virion Structure and Protein Function

SBV has a spherical capsid with icosahedral symmetry with a diameter of 26–31 nm [54,55,56], with plateaus around the fivefold symmetry axis and shallow depressions around the twofold symmetry axes. The major capsid proteins VP1, VP2, VP3, and functional analogs of VP4 subunits make up the capsid, while a minor capsid protein (MiCP), which has not been described in any other *Picornavirales* viruses, has 60 copies attached to the virion surface [53]. When SBV is exposed to an acidic pH during cell entrance, holes with diameters of 7 Å and 12 Å are formed at the threefold and fivefold axes of the capsid, respectively. In contrast, the pores along the twofold icosahedral symmetry axes are currently thought to be the most likely sites for genome release in vertebrate picornaviruses. [53].

Studies relating to SBV have mainly focused on genome sequencing and detection, yet protein function is seldom reported. It has been demonstrated that the VP2 and VP3 proteins have better immunogenicity when compared with VP1 [51] and that VP3 could affect double-stranded RNA (dsRNA) cleavage by inhibiting Dicer enzyme activity and play a role in RNA interference (RNAi) inhibition [57]. The VP1 protein interacts with heat shock protein 70 cognate 5 (Hsp70-c5) and may affect SBV infection [58]. Meanwhile, MiCPs induce liposome disruption, presumably facilitating the passage of the SBV genome into the cytoplasm [53]. Further studies relating to SBV protein function could serve as a robust platform for developing SBV infection prevention and treatment techniques.

### 2.3. Pathogenic Mechanism

Like any other insect, honeybees lack adaptive immunity [59]. However, honeybees show many similarities with vertebrates’ innate immunity, which consists of a sequence of processes, such as the release of antimicrobial peptides (AMPs), phagocytosis, melanization, and pathogen enzymatic destruction [60,61]. SBV infection induces rapid increases in the expression of AMPs, such as *apidaecin*, *hymenoptaecin*, *abaecin*, and *defensin*, which are regulated by Toll and Imd/JNK intracellular pathways [60,62], reflecting the honeybee’s inherent immunity’s ability to create the first line of defense rapidly [63,64]. Other positive responses, including Dicer-like and Argonaute-2 (*Ago2*) genes, which are two core components of RNA interference (RNAi), and bee antiviral protein-1 (Bap-1), have been also found to be significantly upregulated in honeybees in response to SBV infection [65,66]. 

In contrast, Han et al. (2013) confirmed that there are 180 proteins and 19 phosphoproteins with significantly changed expressions in SBV-infected bees, suggesting that the virus disrupts the normal biological processes of honeybees by interfering with carbohydrate metabolism, lipolysis, protein synthesis, the cytoskeletal structure, and immune regulation [67,68]. For instance, SBV infection could downregulate prophenoloxidase (PPO) via the downregulation of upstream signaling serine proteases (SPs) and a prophenoloxidase-activating enzyme (PPAE), as well as the upregulation of serpin, resulting in significant inhibition of the melanization reaction during the immune response [66,69,70]. However, many aspects of host responses to SBV infection are unclear. For example, mechanisms underlying the upregulation of heat shock protein (Hsp) [67], downregulation of tubulin [67,71], alteration of cuticle protein [69,71], and increased triglyceride accumulation [68] still need more experimental verification, and whether these mechanisms can be used as potential therapeutic approaches for controlling SBV infection also needs to be explored.

## 3. Interspecies Transmission

### 3.1. Host Range

In addition to *A. cerana* and *A. mellifera*, SBV has also been detected in *Apis florea*, *Apis dorsata*, and other wild pollinators, such as bumblebees and hoverflies (Table 1). Nonetheless, most of the analyses were only conducted with RT-PCR with the entire insect, and the positive detection of the virus does not necessarily mean infection to the host. Whether SBV could infect these species and what its pathogenicity is need to be further elucidated [72,73,74,75,76].

Related research has uncovered highly prevalent infection rates (the average infection rate is >37%, which goes up to 91.7% in *Bombus* species) in the most common species of the wild bee community [77,78,79]. This frequent spillover between species suggests that the host range of SBV may be much more extensive than originally described [77]. However, the prevalence of SBV in pollinator communities was consistently adversely related to a higher degree of species richness, providing evidence of the dilution effect in viral dilution when SBV infects multiple pollinator host species [80]. Furthermore, the dilution effect differed between hosts, with low-viral-prevalence hosts having more minor dilution effects than high-viral-prevalence counterparts [80]. At the same time, it is essential to note that the more the pollinators that are infected with SBV, the greater the risk of re-infection with SBV among honeybees. Therefore, there is an urgent need to understand this particular virus’s transmission dynamics and assess the risk it represents to pollinator communities.

**Table 1:** Non-*Apis* hymenopteran pollinators that were detected positively with SBV.

Genus/Species	Country/Region	Reference
*Aethina tumida*	America	[79]
*Ancistrocerus auctus*	France	[77]
*Andrena vaga*	Belgium	[81]
*Blattella germanica*	America	[79]
*Bombus atratus*	Colombia	[74]
*Bombus ternarius*	America	[34]
*Bombus terrestris*	France	[77]
*Bombus pascuorum*		
*Bombus ruderatus*		
*Bombus hortorum*	Slovenia	[76]
*Bombus humilis*	France	[77]
*Bombus ignitus*	Korea	[82]
*Bombus impatiens*	America	[80]
*Bombus sylvarum*	Slovenia	[76]
*Bombus vagans*	America	[79]
*Camponotus* spp.		
*Eristalis tenax*	Britain	[83]
*Eristalis arbustorum*		
*Eucera pruinosa*	America	[80]
*Eucera* spp.	France	[77]
*Forficula auricularia*	America	[79]
*Galleria mellonella*		
*Halictidae* sp.	France	[77]
*Halictus fulvipes*		
*Halictus tectus*		
*Hoplitis adunca*		
*Lasioglossum crassepunctatum*		
*Lasioglossum malachurum*		
*Lasioglossum pauperatum*		
*Lasioglossum pauxillum*		
*Megachile albisecta*		
*Nomada distinguenda*		
*Polistes metricus*	America	[34]
*Polistes dominula*	France	[77]
*Scolia flavifrons*		
*Vespula vulgaris*	America	[34]
*Xylocopa violacea*	France	[77]
*Xylocopa virginica*	America	[79]

Note: All the above analyses were conducted with RT-PCR, and only the detections in *Bombus impatiens* and *Eucera pruinosa* were further confirmed with negative-strand RNA detection.

### 3.2. Phylogenetic Classification

SBV has evolved into multiple strains with its various hosts and the different geographic regions it frequents [11,84]. The phylogenetic trees based on the sequences of VP1 [85], RNA-dependent RNA polymerase (RdRp) [11], 5′UTR [47], the entire polyprotein [86], and/or the complete genome [87] have revealed that there are two distinct clusters of SBV: one is composed of the SBV strains from *A. mellifera* (Am genotype or AmSBV), and the other is made up of the SBV strains from *A. cerana* (Ac genotype or AcSBV). Each genotype is further divided into several subtypes according to regional variations [88], such as Korean sacbrood virus (KSBV) [89], Chinese sacbrood virus (CSBV) [90], and Thai sacbrood virus (TSBV) [91] of AcSBV. 

In earlier studies, the SBV strains were revealed to be species specific between *A. mellifera* and *A. cerana* [84,92]. However, in 2016, AcSBV infection was detected in two *A. mellifera* apiaries, causing an approximately 85–90% mortality rate in Linyi City, Shandong Province, China [93]. Using artificial inoculation, Gong et al. (2016) first illustrated that AcSBV is able to cause infection in *A. mellifera*, and they found that 5.26% (2/38) of SBV strains recovered from *A. mellifera* in the field were grouped with *A. cerana* isolates in the phylogenetic tree [11]. Subsequently, Chen et al. (2021) highlighted that 14.28% (4/28) of the total collected SBV isolates from *A. mellifera* were gathered in the AcSBV cluster [45]. From 2017 to 2018, a study in Taiwan showed the AcSBV prevalence rates of *A. cerana* and *A. mellifera* in apiaries, keeping both these two species gradually synchronized [94]. These studies demonstrated that AcSBV is able to infect *A. mellifera* with increasing frequency; in certain circumstances, this cross-species infection of AcSBV may even pose severe threats to the new host.

**Figure 3:**
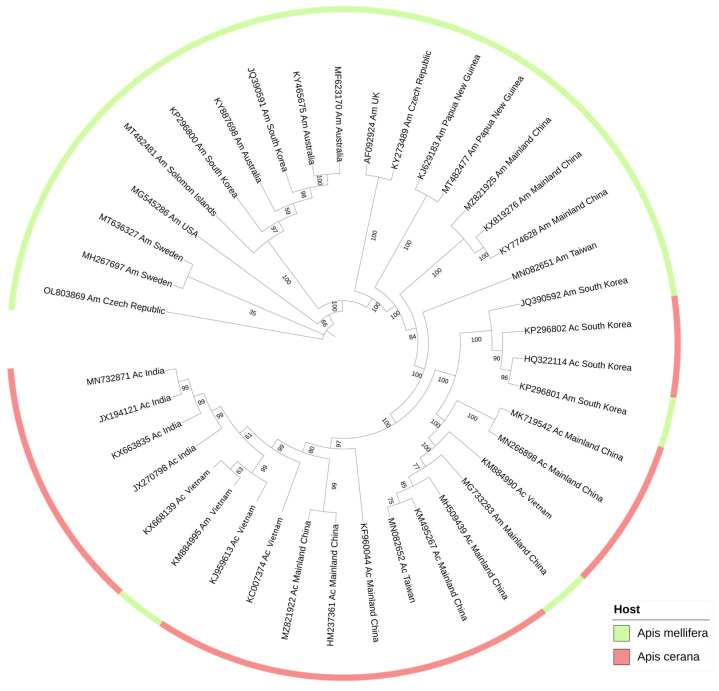
Phylogenetic tree of SBV. This tree was constructed based on the polyprotein sequences of 22 AmSBV and 18 AcSBV strains from the NCBI database. The phylogenetic tree was constructed using the maximum likelihood (ML) method and 1000 bootstrap replications. Strains are annotated to the GenBank accession number, virus host, and region of isolation. Am, *Apis mellifera*; Ac, *A. cerana*. The tree consisted of two branches, mainly AcSBV and AmSBV strains. Nevertheless, the AcSBV cluster also contained four AmSBV isolates from China, South Korea, and Vietnam.

A phylogenetic tree based on polyprotein shows the presence of some AmSBV strains in the cluster of AcSBV, verifying similar conclusions drawn by other studies in the recent years that suggested that there has been cross-infection between *A. cerana* and *A. mellifera* SBV strains (Figure 3). In comparing the entire SBV genomes from the two hosts, the AmSBV strain from the Czech Republic (OL803869) only shared 89.02% identity with the AcSBV strain from India (JX270799); the genome nucleotide identity of AcSBV (MH107056) and AmSBV (KX819276) from China could reach 92.61%. However, the strain from *A. mellifera* in Vietnam (KM884995) was extremely similar to the local AcSBV strain (KJ959613, 99.84%). 

In comparing SBV strains between different regions around the world, the polyprotein nucleotide sequences of AcSBV were highly conserved, sharing more than 90% identity. The AcSBV strain from China (MK719542) was more similar to the strain from Vietnam (KM884990), with 94.59% identity, than to those from South Korea (HQ322114, 93.29%) and India (JX270798, 91.55%). In contrast, the polyprotein nucleotide sequences of AmSBV from the United States (MG545286) shared identities of 89.69% (KM884995, Vietnam) to 97.04% (MT636327, Sweden) with other AmSBV strains.

Comparisons between amino acid sequences revealed that helicase was the most highly conserved region among SBV strains [50], in contrast to the structural protein VP1, which displays the highest number of variations in its amino acid sequence [95]. Unlike most AmSBVs, a portion of amino acids (17 or 10–13) is missing from the VP1 region of AcSBVs [11,50]. Taking advantage of VP1 features, Chang et al. (2021) designed a set of specific primers (sp-SBV-F and sp-SBV-R) that could robustly distinguish between AmSBV and AcSBV strains [96].

In summary, there is cross-infection of SBV strains between *A. mellifera* and *A. cerana*. Considering the high pathogenicity of AcSBV in *A. cerana* colonies, its transmission, and its accumulation in *A. mellifera*, it seems only a matter of time before AcSBV becomes prevalent among wild pollinators and causes population losses in the future. This suggests that further investigation is urgently needed, especially for the detection and prevalence of AcSBV in *A. mellifera* and other pollinators and the extent of population damage it causes worldwide.

Considering the quasispecies nature of RNA viruses due to their high mutation rate, the genetic variation of SBV in *A. mellifera* and *A. cerana* is worth further investigation to enhance our understanding of the virus’s evolution and the connectivity between the quasispecies dynamics and viral pathogenesis.

## 4. Diagnostic Method

### 4.1. Clinical Diagnosis 

SBV is one of the few honeybee viruses that can cause apparent disease symptoms. The distinctive disease symptoms described in Section 1.1 can be used for identifying sacbrood disease in the field. However, the disease’s symptoms look similar to those of other brood diseases. American foulbrood (AFB) and European foulbrood (EFB) are worldwide-distributed honeybee brood diseases that are caused by the bacteria *Paenibacillus larvae* and *Melissococcus plutonius*, respectively [97,98]. The significant differences between sacbrood disease and AFB/EFB are that unlike AFB/EFB, the SBV-infected brood does not die in the pupal stage nor decompose and the dead larvae are odorless after SBV infection [20,22,99]. However, co-infection of pathogens is common in honeybees, meaning the symptoms can be ambiguous when several causative agents exist. Moreover, as with other bee viruses, asymptomatic infections with SBV are prevalent. Laboratory tests are thus needed when there are inapparent or no characteristic symptoms in the colony.

### 4.2. Laboratory Identification Methods

Laboratory-based detection methods for SBV include electron microscope identification, serological assays, and nucleic-acid-based detection approaches. However, electron microscopy is not commonly used for routine diagnostic submissions of SBV infection due to it being an expensive and time-consuming procedure. Even during the virion observation in the laboratory, this method should be used with great care and meticulousness because of the similarities with regard to virion morphology between SBV and other honeybee viruses and the prevalence of viral co-infection in honeybees [53].

Conversely, enzyme-linked immunosorbent assay (ELISA) is the preferred detection method for large samples. In addition to the purified SBV virion, the recombinant proteins rVP1, rVP2, and rVP3 have been proven to have good immunogenicity with monoclonal and polyclonal antibody production [51,100,101]. Nevertheless, ELISA often lacks the appropriate level of resolution to appropriately identify viral strains, which have also been the focus of recent research on SBV trans-species transmission; this frequently occurs because the strain-determining characteristic is not represented in a coat protein variation or because the viral coat proteins are so highly conserved within a genus that antibodies cannot be used to discriminate between the strains [102].

Molecular biology has revolutionized the diagnosis of bee diseases. Many nucleic-acid-based molecular methods are used to detect SBV in laboratories these days. Reverse transcription-polymerase chain reaction (RT-PCR) [103] and quantitative RT-PCR (qRT-PCR) [104] or other approaches based on the extension of these two detection methods, such as multiplex RT-PCR [103], are commonly used methods for SBV detection in laboratories [105,106,107,108]. Most primers (Table S2) have been designed based on the conserved region of SBV strains. For example, the primer pair designed by Sguazza et al. (2013) was based on the conserved regions of various strains from diverse regions worldwide; this primer pair was able to align with most SBV strains with genomic sequences deposited in the NCBI (99 of 100 strains achieved 100% match) [109]. In addition, traditional PCR-based assays require the sacrifice of live bees to homogenize tissue and then perform nucleic acid isolation. A non-sacrificial approach for obtaining a tiny volume of hemolymph was recently devised, which can be used for detection directly without the involvement of nucleic acid extraction and a molecular analysis can be performed immediately [104]. 

Moreover, another class of detection method we want to mention is loop-mediated isothermal amplification (LAMP) [110,111]; its modes of detection and diagnosis can be transferred to a poorly resourced laboratory, or they could even be undertaken in the field [102], which is also required for global prevalence surveys of SBV. As an alternative, helicase-dependent amplification (HDA) requires fewer primers than LAMP to complete the diagnosis under similar experimental conditions; nevertheless, its success rate in detecting SBV has not been widely reported [112].

## 5. Prevention and Control

### 5.1. Colony Management

Up to now, no chemical therapy has been specifically designed to target SBV. Due to the risk of drug residues in honeybee products, the frequency of the use of chemical drugs should be limited to as little as possible. Preventing and controlling sacbrood disease through colony management is thus the first strategy that needs to be considered and proven effective [113]. For instance, removing extra combs during routine management to maintain an equilibrium between the number of combs and honeybee workers helps prevent and control honeybee diseases in general [114]. If the disease has already occurred, removing combs filled with the diseased brood and sterilizing the honeycombs and hives will reduce the horizontal transmission of the virus within the colony. In light of the vertical transmission of the virus from the queen to her brood, replacing the queen with a young and healthy queen or caging the queen to prevent her from laying eggs for 10–14 days usually has a substantial positive effect on healing the colony [115]. In addition, the colony’s nutritional status is another factor that needs to be considered and addressed. For example, a survey conducted in Serbia confirmed that a plant-based supplement containing B-complex vitamins considerably lowers the infection loads of SBV [116].

### 5.2. RNA Interference

In eukaryotes, RNAi is an evolutionarily conserved post-transcriptional gene-silencing process [117]. The primary antiviral response of honeybees is small interfering RNA (siRNA) in three distinct pathways of RNAi [118]. The dsRNA synthesized during viral replication is recognized by the host endoribonuclease Dicer and cleaved into siRNAs; these siRNAs can then mediate the cleavage of homologous viral genomic RNA [118]. Indeed, it has been proven possible to treat a viral infection by introducing exogenous dsRNA into the insects’ cells to activate RNAi pathways [119]. Furthermore, it has been confirmed that the RNAi immune response is triggered by Dicer-2 when honeybees are infected with SBV [118,120].

RNAi has been shown as a quick and highly effective strategy for protecting honeybees against viral infections [121]. Virus replication was considerably suppressed when honeybee larvae were given dsRNA matching CSBV’s capsid protein VP1 [122]. Furthermore, another study showed that RNAi treatment begins to affect the larvae of *A. cerana* infected with CSBV 12 h after the oral application of dsRNA [123]. Zhang et al. (2016) fed 12 honeybee colonies the SBV dsRNA produced by *Escherichia coli* (*E. coli*) HT115 (DE3) during field application based on the experiment, and the infection rate dropped by 40.66% compared to the colony only fed with CSBV; this finding suggests a strategy for producing and purifying the dsRNA of SBV on a large scale [122]. However, because of its high cost [124], potential off-target effects, and the risk of residues being found in bee products [125], the application of RNAi in honeybee disease prevention has been restricted.

### 5.3. Antibody Treatment

Egg yolk antibodies have been used to treat viral diseases, such as the porcine epidemic diarrhea virus [126] and rotavirus [127], and have exhibited significant therapeutic effects. Unlike vertebrates, honeybees do not have acquired immunity as an antiviral route for lacking antibodies after a viral infection. In response, Sun et al. (2018) produced a specific IgY via the immunization of white leghorn hens with inactivated CSBV. Antibodies to the yolk were discovered to have a considerable effect on CSBV in both laboratory and field tests. According to the findings, “universal” passive immunotherapy by using a particular IgY for CSBV might be a novel way to control CSBV infection [128].

### 5.4. Herbal Medicines

Plant extracts exhibit various pharmacological antibiotic, antiviral, and anti-inflammatory effects. Water extractions of some Chinese herbal medicines were reported to be effective in controlling sacbrood disease during field tests (Table 2). Nonetheless, among all the materials used, only indigowoad root (*Radix isatidis*) was experimentally confirmed in the laboratory to inhibit the replication of SBV, reduce the expression of antimicrobial peptides, and significantly increase the survival rate of artificially infected *A. cerana* larvae (from 43% to 93%) [63]. Although some studies suggest that using multiple herbs in combination can significantly reduce the prevalence of CSBV and the mortality of infected larvae, their specific active components, mechanisms, and therapeutic effects remain to be further studied.

**Table 2:** Chinese herbal medicines reported to be effective in controlling sacbrood disease in *A. cerana* in China.

Herb	References
Indigowoad Root (*Radix Isatidis*)	[129,130,131,132]
Cyrtomium Rhizome (*Rhizoma Cyrtomii*)	[133,134,135,136,137]
Honeysuckle Flower (*Flos Lonicerae*)	[130,133,137,138]
Barbed Skullcap Herb (*Herba Scutellariae Barbatae*)	[87,129,130]
Liquorice Root (*Radix Glycyrrhizae*)	[131,135,137,138]
Polygoni Cuspidati Rhizoma (*Polygonum Cuspidatum*)	[130,133,136]
Mongolian Dandelion Herb (*Herba Taraxaci*)	[135,137,139]
Slender Dutchmanspipe Root (*Radix Aristolochiae*)	[131,135,136]
Cassia Twig (*Ramulus Cimnamomi*)	[129,131,137]
Pericarpium Papaveris (*Papaver somniferum*)	[133,135,136]

Note: Only the effect of indigowoad root (*Radix isatidis*) was experimentally confirmed in the laboratory.

## 6. Conclusions

As a globally prevalent honeybee pathogen, sacbrood virus is of great concern to agronomists and ecologists who realize that SBV strains among new hosts are persistently emerging. Even though some genotypes are restricted to specific geographic regions, AcSBV strains that have already infected *A. mellifera* colonies could be widely spread by ectoparasitic mites, pollen, and the movement of the honeycomb and other hive products. Considering the high pathogenicity of AcSBV strains in *A. cerana*, global pollinators may be facing a significant threat to their existence. Therefore, it is suggested that a greater commitment to the detection and sequencing of SBV, which can infect honeybees and other species worldwide, is vital. There is also a need to develop cheaper, quick, and energy-efficient detection methods and low-cost, definitive curative medicines that can be mass-produced to aid the treatment of SBV infections in honeybees.

## Supplementary Materials

The following are available online at https://www.mdpi.com/article/10.3390/v14091871/s1, Table S1: Information of Sacbrood virus prevalence detection, Table S2: Primer list for SBV detection. References cited in the supplementary tables are included in the reference list [140,141,142,143,144,145,146,147,148,149,150,151,152,153,154,155,156,157,158,159,160,161,162,163,164,165,166,167,168,169,170,171,172,173,174,175,176,177,178,179,180,181,182,183,184,185,186,187,188,189,190,191,192,193,194,195].
