# Supplementary material for: Sacbrood Virus: A Growing Threat to Honeybees and Wild Pollinators
Source: Viruses. 2022 Aug 25;14(9):1871. doi: 10.3390/v14091871 (PMC9505205; doi:10.3390/v14091871)
Supplement: Supplementary file 1 [file viruses-14-01871-s001.zip › viruses-1791653-Supplementary.pdf]

Table S1. Information of Sacbrood virus prevalence detection. The primer sequences are shown in Table S2.

| Number | Region | Year | Host | Prevalence | Sample size | Primer number | NCBI Accession | Reference |
|---|---|---|---|---|---|---|---|---|
| 1 | Algeria | 2013 | AM | 13.79% | 29 | 2 | - | [140] |
| 2 | Argentina | 2015 | AM | 1.10% | 385 | 3 | - | [141] |
| 3 | Australia | 2015 | AM | 35% | 1240 | 9 | - | [142] |
| 4 | Austria | 2004 | AM | 49% | 90 | 1 | - | [143] |
| 5 | Bangladesh | 1995 | * | + | * | - | - | [144] |
| 6 | Belarus | 1996 | * | + | * | - | - | [72] |
| 7 | Belgium | 2017 | AM | 97.13% | 557 | 8 | - | [145] |
| 8 | Belize | 1993 | * | + | * | - | - | [146] |
| 9 | Bhutan | 1990 | * | + | * | - | - | [14] |
| 10 | Brazil | 2011 | AM | 18.18% | 11 | 12 | - | [8] |
| 11 | Bulgaria | 2018 | AM | 12% | 50 | 2 | MG649495-99 | [147] |
| 12 | Canada | 2010 | AM | 16% | 103 | 6 | - | [148] |
| 13 | Chile | 2010 | AM | 14.29% | 21 | 4 | - | [149] |
| 14 | China | 2019 | AC | 20% | 15 | 1 | MW892515-24 | [150] |
| 15 | Colombia | 2014 | AM | 23.40% | 483 | 4 | - | [151] |
| 16 | Costa Rica | 1996 | * | + | * | - | - | [152] |
| 17 | Croatia | 2010 | AM | 40.24% | 82 | 1 | - | [153] |
| 18 | Cuba | 2019 | AM | 9.09% | 22 | 11 | - | [154] |
| 19 | Cyprus | 1996 | * | + | * | - | - | [72] |
| 20 | Czech Rep. | 2017 | AM | 3.03% | 33 | 1 | MK550481 | [32] |
| 21 | Denmark | 2012 | AM | 39% | 269 | 1 | - | [155] |
| 22 | Ecuador | 2017 | AM | 11.76% | 17 | 5 | - | [156] |
| 23 | Egypt | 1988 | * | + | * | - | - | [157] |
| 24 | Fiji | 1986 | AM | 79% | 96 | - | - | [158] |
| 25 | Finland | 1993 | * | + | * | - | - | [146] |
| 26 | France | 2015 | AC | 91.30% | 23 | 5 | - | [77] |
| 27 | Germany | 2017 | AM | 11.10% | 1064 | 3 | - | [159] |
| 28 | Greece | 2011 | AM | 6.67% | 15 | 6 | - | [160] |
| 29 | Guatemala | 1996 | * | + | * | - | - | [152] |
| 30 | Honduras | 1993 | * | + | * | - | - | [146] |

| Number | Region | Year | Host | Prevalence | Sample size | Primer number | NCBI Accession | Reference |
|---|---|---|---|---|---|---|---|---|
| 31 | Hungary | 2004 | AM | 2% | 52 | 1 | - | [161] |
| 32 | India | 2008 | AM | 0.08%-14.70% | * | - | - | [162] |
| 33 | Indonesia | 2014 | AC | + | * | 1 | KJ629177 | [88] |
| 34 | Iran | 2012 | AM | 18.50% | 160 | 1 | - | [163] |
| 35 | Ireland | 1993 | * | + | * | - | - | [146] |
| 36 | Israel | 2009 | AM | 4% | 71 | * | - | [164] |
| 37 | Italy | 2021 | AM | 100% | 180 | 1 | - | [165] |
| 38 | Japan | 2018 | AM | 3.80% | 26 | 1 | - | [166] |
| 39 | Jordan | 1993 | * | + | * | - | - | [157] |
| 40 | Kenya | 2014 | AM | 14% | 100 | 1 | - | [167] |
| 41 | Korea | 2012 | AC | 35.29% | 527 | 1 | JN542440-42 | [168] |
| 42 | Latvia | 1995 | * | + | * | - | - | [144] |
| 43 | Lithuania | 2014 | AM | 33.70% | 359 | 6 | KP223782-88 | [169] |
| 44 | Luxembourg | 2011 | AM | 100% | 20 | 3 | - | [170] |
| 45 | Malaysia | 2014 | AC | + | * | 1 | KJ629178 | [88] |
| 46 | Mexico | 2012 | AM | 20% | 10 | 2 | - | [171] |
| 47 | Moldova | 1996 | * | + | * | - | - | [72] |
| 48 | Mongolia | 2013 | AM | 73.50% | 151 | 3 | LN875562-68 | [172] |
| 49 | Morocco | 2018 | AM | 14.20% | 60 | 2 | MT863272 | [173] |
| 50 | Myanmar | 1993 | * | + | * | - | - | [146] |
| 51 | Nepal | 1999 | AM | + | * | 1 | AF284616-29 | [174] |
| 52 | New Zealand | 2002 | AM | 5.5%-15% | 20 | - | - | [175] |
| 53 | Nicaragua | 1996 | * | + | * | - | - | [152] |
| 54 | Niue | 1993 | * | + | * | - | - | [146] |
| 55 | Norway | 2014 | AM | + | * | 8 | - | [176] |
| 56 | Oman | 1997 | * | + | * | - | - | [157] |
| 57 | Pakistan | 1996 | * | + | * | - | - | [72] |
| 58 | Panama | 1996 | * | + | * | - | - | [152] |
| 59 | Papua New Guinea | 2014 | AM | + | * | 1 | KJ629175-76 | [88] |
| 60 | Peru | 2014 | AM | 0% | 10 | * | - | [177] |
| 61 | Philippines | 1996 | AC | + | 37 | - | - | [178] |

| Number | Region | Year | Host | Prevalence | Sample size | Primer number | NCBI Accession | Reference |
|---|---|---|---|---|---|---|---|---|
| 62 | Poland | 2004 | AM | + | 12 | 1 | - | [143] |
| 63 | Romania | 1993 | * | + | * | - | - | [146] |
| 64 | Russia | 2016 | AM | + | * | * | * | [179] |
| 65 | Samoa | 1993 | * | + | * | - | - | [146] |
| 66 | Saudi Arabia | 1990 | * | + | * | - | - | [157] |
| 67 | Serbia | 2017 | AM | 24% | 150 | 7 | KM001901 | [180] |
| 68 | Slovenia | 2020 | AM | 31.48% | 305 | 9 | - | [181] |
| 69 | Solomon Islands | 1993 | * | + | * | - | - | [146] |
| 70 | South Africa | 1999 | AM | + | * | 1 | AF284616-29 | [174] |
| 71 | Spain | 2008 | AM | 0.83% | 484 | 4 | FJ812813-14 | [108] |
| 72 | Sweden | 2009 | AM | + | 25 | 3 | - | [182] |
| 73 | Switzerland | 2008 | AM | 7.7%-43.8% | 29 | 2 | - | [183] |
| 74 | Syria | 2017 | * | 13.33% | 240 | * | - | [184] |
| 75 | Thailand | 2007 | AM | 15.22% | 46 | 2 | - | [185] |
| 76 | Tonga | 1996 | * | + | * | - | - | [72] |
| 77 | Trinidad and Tobago | 1995 | * | + | * | - | - | [144] |
| 78 | Tunisia | 1988 | * | + | * | - | - | [157] |
| 79 | Turkey | 2018 | AM | 22.30% | 76 | 5 | - | [186] |
| 80 | Uganda | 2010 | AM | 0% | 138 | 7 | - | [187] |
| 81 | Ukraine | 2016 | AM | + | * | 1 | - | [188] |
| 82 | United Kingdom | 2006 | AM | 1.40% | 69 | 2 | DQ434992 | [189] |
| 83 | United States | 2016 | AM | 12%-100% | 50 | 1 | - | [46] |
| 84 | Uruguay | 2011 | AM | 19.40% | 103 | 4 | - | [190] |
| 85 | Venezuela | 1993 | * | + | * | - | - | [146] |
| 86 | Viet Nam | 2015 | AC | 18.33% | 180 | 1 | - | [13] |
| 87 | Yemen | 2017 | AM | 0% | 16 | * | - | [191] |

AC: *Apis cerana*; AM: *Apis mellifera*; *: Absence of record; -: No information available; +: Present.

Table S2. Primer list for SBV detection.

| Primer number | Sequence (5′ to 3′) | Reference sequence | Nucleotide positions | Length (bp) | Reference |
|---|---|---|---|---|---|
| 1 | F: ACC AAC CGA TTC CTC AGT AG | AF092924 | 221-240 | 487 | [174] |
| | R: CCT TGG AAC TCT GCT GTG TA | | 689-708 | | |
| 2 | F: GGA TGA AAG GAA ATT ACC AG | AF092924 | * | 426 | [192] |
| | R: CCA CTA GGT GAT CCA CAC T | | * | | |
| 3 | F: TTG GAA CTA CGC ATC TCT G | * | 3164-3182 | 335 | [193] |
| | R: GCT CTA ACC TCG CAT CAA C | | 3461-3479 | | |
| 4 | F: CGA GTG TTG TGT GTG TAA AGA GA | NC002066 | 5307-5329 | 341 | [108] |
| | R: CGA AGG GTG AAG TGT AGC AG | | 5628-5647 | | |
| 5 | F: CGT AAT TGC GGA GTG GAA AGA TT | AF092924 | 314–336 | 342 | [109] |
| | R: AGA TTC CTT CGA GGG TAC CTC ATC | | 655–632 | | |
| 6 | F: GCT GAG GTA GGA TCT TTG CGT | AF092924 | 4957-4977 | 825 | [194] |
| | R: TCA TCA TCT TCA CCA TCC GA | | 5762-5781 | | |
| 7 | F: GCA CGT TTA ATT GGG GAT CA | AF092924 | 1655-1674 | 693 | [34] |
| | R: CAG GTT GTC CCT TAC CTC CA | | 2328-2347 | | |
| 8 | F: GGG TCG AGT GGT ACT GGA AA | AF092924 | * | * | [195] |
| | R: ACA CAA CAC TCG TGG GTG AC | | * | | |
| 9 | F: AAC GTC CAC TAC ACC GAA ATG TC | AF092924 | 434-456 | 70 | [176] |
| | R: ACA CTG CGC GTC TTA ACA TTC C | | 482-503 | | |
| | P: TGA TGA GAG TGG ACG AAG A | | 460-478 | | |
| 10 | F: AGC CAG GTG ATA GAT GCT C | * | 5030-5048 | 338 | [181] |
| | R: AAA TAC TCC CGC CAA ATC AC | | 5349-5368 | | |
| | P: (6-Fam) TGG CTC ATC TGG GAT CAC AAT TTC C (Tamra) | | * | | |

F, Forward; R, Reverse; P: Probe; *: Absence of record.
